# The Disinfection and Sterilization of Surgical Instruments

**Published:** 1892-07

**Authors:** 


### THE DISINFECTION AND STERILIZATION OF SURGICAL INSTRUMENTS.

According to Maljean, the best method of destroying pyogenic and infecting germs, whose resistance is increased when enveloped in a dried albuminoid, as blood or pus, is to boil all metallic instruments for twenty minutes in a solution of sodium carbonate, 1 per cent. being as efficient as a stronger solution, as it acts not as a germicide but as a dissolvent of the dried albuminoids, and allows the heat to act upon the spores. The cleaning of the instruments with soap and brush is not to be discontinued, but they are to be cleaned before being boiled; and as an efficient method of preventing the drying upon instruments, of pus and blood, he advises the placing of them, immediately after use, in a warm solution of soda that has been previously boiled for ten minutes. For instruments not entirely metallic, as knives, he advises the dipping of the blades in a boiling 5-per-cent. carbolic solution for five minutes, the whole instrument to be placed in a cold solution for fifteen minutes. He found that the spores of vibrio septicæmiæ and tetanus bacillus, when covered by dried albuminoids, were unaffected by remaining in a

5-per-cent. solution of carbolic acid for five days, and that at the end of four days they were living in a neutral 1:1000 sublimate solution, and concludes that there is no practical antiseptic capable of destroying germs in a sufficiently short time without the addition of heat.

[It may fairly be said that this method of boiling in soda solution is that preferred and employed by the great majority of surgeons at the present day, because of its convenience and certainty of action. If, however, for any reason it is not possible to employ it, immersion of the instruments for twenty minutes in a 1:20 carbolic solution, or for a much shorter time in a 1:1000 sublimate solution, will render them aseptic in the great majority of cases.]—*Amer. Journ. Med. Science.*

